# Supplementary material for: a-Synuclein and lipids in erythrocytes of Gaucher disease carriers and patients before and after enzyme replacement therapy
Source: PLoS One. 2023 Feb 3;18(2):e0277602. doi: 10.1371/journal.pone.0277602 (PMC9897572; doi:10.1371/journal.pone.0277602)
Supplement: S1 Table — (DOCX) [file pone.0277602.s001.docx]

**S1 Table. Genotypes of the Gaucher disease patients and carriers**

**studied.**

| **Gr A**  n=45 | | | **Gr C**  n=19 | |
| --- | --- | --- | --- | --- |
| **Genotype** | **n** | **GD Type** | **Genotype** | **n** |
| N370S/N370S | 7 | I | D409H:H255Q/wt | 7 |
| N370S/D409H:H255Q | 14 | I | N370S/wt | 6 |
| N370S/L444P | 2 | I | L444P/wt | 2 |
| N370S/IVS6-2A>G | 3 | I | IVS10-1G>A/wt | 1 |
| N370S/RecNciI | 2 | I | G202R/wt | 1 |
| N370S/IVS10-1G>A | 5 | I | D409H/wt | 2 |
| N370S/R120W | 2 | I |  |  |
| N370S/G202R | 1 | I |  |  |
| N370S/Y135S | 1 | I |  |  |
| N370S/L175P | 1 | I |  |  |
| L444P/F81L | 1 | I |  |  |
| L444P/Y116H | 1 | I |  |  |
| D409H:H255Q/D409H:H255Q | 1 | II |  |  |
| D409H:H255Q/R120W | 1 | II |  |  |
| D409H:H255Q/Y108C | 1 | III |  |  |
| D409H:H255Q/L444P | 1 | III |  |  |
| D409H/L444P | 1 | III |  |  |

GrA, Gaucher disease patients receiving no treatment; GrC, Gaucher disease carriers; n, number of individuals. RecNciI (L444P, A456P, V460V);

IVS10-1G>A (c.[1505+1_1505+12ins;1505G>A]).
