# Supplementary material for: a-Synuclein and lipids in erythrocytes of Gaucher disease carriers and patients before and after enzyme replacement therapy
Source: PLoS One. 2023 Feb 3;18(2):e0277602. doi: 10.1371/journal.pone.0277602 (PMC9897572; doi:10.1371/journal.pone.0277602)
Supplement: S2 Table — (DOCX) [file pone.0277602.s002.docx]

**S2 Table.** **Percentage of Galactosylsphingosine (GalSph) and Glucosylsphingosine (GlcSph) detected following the separation of the Glucose- from the Galactose-containing Hexosylsphingosine (HexSph) species using a HILIC column.**

| Sample ID | % GalSph | % GlcSph |
| --- | --- | --- |
| 1 | 1 | 99 |
| 2 | 2 | 98 |
| 3 | 3 | 97 |
| 4 | 23 | 77 |
| 5 | 15 | 85 |
| 6 | 9 | 91 |
| Average | **9** | **91** |
