# Supplementary material for: a-Synuclein and lipids in erythrocytes of Gaucher disease carriers and patients before and after enzyme replacement therapy
Source: PLoS One. 2023 Feb 3;18(2):e0277602. doi: 10.1371/journal.pone.0277602 (PMC9897572; doi:10.1371/journal.pone.0277602)
Supplement: S3 Table — (DOCX) [file pone.0277602.s003.docx]

**S3 Table. Red blood cell membrane levels and statistical comparison of the lipids studied in Gaucher**

**disease patients receiving no treatment, Gaucher disease carriers and controls.**

|  | **GrA**  n=45 | **GrC**  n=19 | **GrD**  n=49 | **p-value** | | |
| --- | --- | --- | --- | --- | --- | --- |
|  |  |  |  | **A/C** | **A/D** | **C/D** |
| **C16:0 - GlcCer**  (pmoles/10^8^ cells) | median: 14.90  range: 5.98-56.70 | median: 6.21  range: 3.98-16.13 | median: 6.75  range: 4.41-11.58 | <0.001* | <0.001* | 0.282 |
| **C18:0 - GlcCer**  (pmoles/10^8^ cells) | median: 0.33  range: 0.14-0.81 | median: 0.21  range: 0.12-0.47 | median: 0.19  range: 0.12-0.38 | <0.001* | <0.001* | 0.233 |
| **C24:1 - GlcCer**  (pmoles/10^8^ cells) | median: 0.74  range: 0.31-4.28 | median: 0.58  range: 0.29-2.10 | median: 0.61  range: 0.30-1.43 | 0.007* | 0.027* | 0.151 |
| **SUM - GlcCer**  (pmoles/10^8^ cells) | median: 15.93  range: 6.91-59.51 | median: 7.10  range: 4.58-17.24 | median: 7.62  range: 4.95-12.75 | <0.001* | <0.001* | 0.233 |
| **C16:0 DMA/C16:0** | median: 0.091  range: 0.045-0.131 | median: 0.106  range: 0.075-0.124 | median: 0.107  range: 0.085-0.145 | <0.001* | <0.001* | 0.341 |
| **C18:0 DMA/C18:0** | median: 0.179  range: 0.086-0.219 | median: 0.203  range: 0.153-0.232 | median: 0.200  range: 0.160-0.249 | <0.001* | <0.001* | 0.300 |
| **GlcChol**  (pmoles/10^8^ cells) | median: 10.27  range: 6.21-17.69 | median: 6.98  range: 4.26-10.85 | median: 6.57  range: 4.94-11.36 | <0.001* | <0.001* | 0.081 |
| **HexSph**  (pmoles/10^8^ cells) | median: 14.40  range: 0.57-82.26 | median: 0.062  range: 0.033-0.153 | median: 0.047  range: 0.017-0.184 | <0.001* | <0.001* | 0.066 |

C16:0-GlcCer, N-palmitoyl-glucosylceramide; C18:0-GlcCer, N-stearoyl-glucosylceramide; C24:1-GlcCer, N-nervonoyl-glucosylceramide; SUM-GlcCer, sum of glucosylceramide species; C16:0 DMA/C16:0, C16:0-plasmalogens; C18:0 DMA/C18:0, C18:0-plasmalogens; GlcChol, glucosylcholesterol; HexSph, hexosylsphingosine; GrA, Gaucher disease patients receiving no treatment; GrC, Gaucher disease carriers**;** GrD, controls.

**Statistically significant differences*
