# Supplementary material for: a-Synuclein and lipids in erythrocytes of Gaucher disease carriers and patients before and after enzyme replacement therapy
Source: PLoS One. 2023 Feb 3;18(2):e0277602. doi: 10.1371/journal.pone.0277602 (PMC9897572; doi:10.1371/journal.pone.0277602)
Supplement: S4 Table — (DOCX) [file pone.0277602.s004.docx]

**S4 Table. Red blood cell membrane levels and statistical comparison of the α-synuclein species**

**studied in Gaucher disease patients receiving no treatment, Gaucher disease carriers and controls.**

|  | **GrA**  n=45 | **GrC**  n=19 | **GrD**  n=49 | **p-value** | | |
| --- | --- | --- | --- | --- | --- | --- |
|  |  |  |  | **A/C** | **A/D** | **C/D** |
| **α-Syn Monomer** | median: 1.27  range: 0.27-2.13 | median: 1.12  range: 0.25-1.50 | median: 1.02  range: 0.04-2.46 | 0.149 | 0.023* | 0.320 |
| **α-Syn Dimer** | median: 1.39  range: 0.52-9.41 | median: 1.32  range: 0.49-3.39 | median: 1.02  range: 0.05-3.83 | 0.180 | 0.001* | 0.075 |
| **Dimer/Monomer**  **Ratio** | median: 1.28  range: 0.62-8.56 | median: 1.30  range: 0.76-2.58 | median: 0.91  range: 0.17-4.25 | 0.460 | 0.005* | 0.019* |

GrA, Gaucher disease patients receiving no treatment; GrC, Gaucher disease carriers**;** GrD, controls.

**Statistically significant differences*
