# Supplementary material for: a-Synuclein and lipids in erythrocytes of Gaucher disease carriers and patients before and after enzyme replacement therapy
Source: PLoS One. 2023 Feb 3;18(2):e0277602. doi: 10.1371/journal.pone.0277602 (PMC9897572; doi:10.1371/journal.pone.0277602)
Supplement: S5 Table — (DOCX) [file pone.0277602.s005.docx]

**S5 Table. Red blood cell membrane levels and statistical comparison of the lipids studied**

**in Gaucher disease patients before and after ERT.**

|  | **GrA1**  n=13 | **GrΒ**  n=13 | **GrD**  n=49 | **p-value** | |
| --- | --- | --- | --- | --- | --- |
|  |  |  |  | **A1/B** | **B/D** |
| **C16:0 - GlcCer**  (pmoles/10^8^ cells) | median: 15.16  range: 8.33-19.48 | median: 8.03  range: 4.96-23.01 | median: 6.75  range: 4.41-11.58 | <0.001* | 0.154 |
| **C18:0 - GlcCer**  (pmoles/10^8^ cells) | median: 0.35  range: 0.19-0.50 | median: 0.22  range: 0.10-0.77 | median: 0.19  range: 0.12-0.38 | 0.094 | 0.098 |
| **C24:1 - GlcCer**  (pmoles/10^8^ cells) | median: 0.83  range: 0.34-2.19 | median: 0.56  range: 0.33-4.79 | median: 0.61  range: 0.30-1.43 | 0.168 | 0.256 |
| **SUM - GlcCer**  (pmoles/10^8^ cells) | median: 16.43  range: 9.19-21.14 | median: 8.69  range: 5.48-28.57 | median: 7.62  range: 4.95-12.75 | 0.006* | 0.209 |
| **C16:0 DMA/C16:0** | median: 0.088  range: 0.054-0.120 | median: 0.106  range: 0.086-0.122 | median: 0.107  range: 0.085-0.145 | 0.036* | 0.305 |
| **C18:0 DMA/C18:0** | median: 0.181  range: 0.101-0.219 | median: 0.196  range: 0.174-0.212 | median: 0.200  range: 0.160-0.249 | 0.162 | 0.095 |
| **GlcChol**  (pmoles/10^8^ cells) | median: 10.42  range: 8.35-14.62 | median: 8.06  range: 3.54-11.40 | median: 6.57  range: 4.94-11.36 | <0.001* | 0.006* |
| **HexSph**  (pmoles/10^8^ cells) | median: 18.41  range: 6.58-44.35 | median: 3.42  range: 0.59-10.64 | median: 0.047  range: 0.017-0.184 | <0.001* | <0.001* |

C16:0-GlcCer, N-palmitoyl-glucosylceramide; C18:0-GlcCer, N-stearoyl-glucosylceramide; C24:1-GlcCer, N-nervonoyl-glucosylceramide; SUM-GlcCer, sum of glucosylceramide species; C16:0 DMA/C16:0, C16:0-plasmalogens; C18:0 DMA/C18:0, C18:0-plasmalogens; GlcChol, glucosylcholesterol; HexSph, hexosylsphingosine; GrA1, Gaucher disease patients receiving no treatment; GrB, the GrA1 Gaucher disease patients following one year of ERT; GrD, controls.

**Statistically significant differences*
