# Supplementary material for: a-Synuclein and lipids in erythrocytes of Gaucher disease carriers and patients before and after enzyme replacement therapy
Source: PLoS One. 2023 Feb 3;18(2):e0277602. doi: 10.1371/journal.pone.0277602 (PMC9897572; doi:10.1371/journal.pone.0277602)
Supplement: S6 Table — (DOCX) [file pone.0277602.s006.docx]

**S6 Table. Red blood cell membrane levels and statistical comparison of the α-Synuclein**

**species studied in Gaucher disease patients before and after ERT.**

|  | **GrA1**  n=13 | **GrB**  n=13 | **GrD**  n=49 | **p-value** | |
| --- | --- | --- | --- | --- | --- |
|  |  |  |  | **A1/B** | **B/D** |
| **α-Syn Monomer** | median: 0.93  range: 0.43-1.59 | median: 1.03  range: 0.42-1.75 | median: 1.02  range: 0.04-2.46 | 0.842 | 0.390 |
| **α-Syn Dimer** | median: 1.24  range: 0.62-4.42 | median: 1.28  range: 0.02-3.60 | median: 1.02  range: 0.05-3.83 | 0.125 | 0.222 |
| **Dimer/Monomer**  **Ratio** | median: 1.70  range: 0.62-4.47 | median: 1.16  range: 0.04-2.40 | median: 0.91  range: 0.17-4.25 | 0.011* | 0.433 |

GrA1, Gaucher disease patients receiving no treatment; GrB, the GrA1 Gaucher disease patients following one year of ERT; GrD, controls.

**Statistically significant differences*
